# Pushing Past Limits: How Efficacious Is High-Effort Coping for Self-Rated Health among African American and Caribbean Black Women?

**DOI:** 10.3390/ijerph192013460

**Published:** 2022-10-18

**Authors:** Millicent N. Robinson

**Affiliations:** School of Social Work, University of North Carolina at Chapel Hill, Chapel Hill, NC 27599, USA; millinr2@unc.edu

**Keywords:** health-protective mechanisms, heterogeneity within groups, coping, self-rated health, Black women, Caribbean Black women, John Henryism

## Abstract

Due to systemic oppression, Black women experience distinct risks across the life course, such as exposure to various stressors that shape lower ratings of self-rated health. This is important given that self-rated health is a stronger indicator of current morbidity and subsequent mortality than physician assessments. However, there has been limited consideration of the role of coping in shaping self-rated health among this group. John Henryism, or high-effort coping, is a culturally relevant coping style that reflects the broader societal, cultural, and historical context that shapes lived experiences of Black populations navigating racism and capitalism in the U.S., and has received limited consideration in health research among Black women. Additionally, less is known regarding how ethnicity shapes John Henryism and health processes among Black women specifically. Therefore, the present study examined the association between John Henryism and self-rated health among African American and Caribbean Black women (*n* = 1580) collectively, and explored this association among Caribbean Black women specifically, utilizing the National Survey of American Life (NSAL 2001–2003). Findings show that while John Henryism was not directly associated with self-rated health among either group, once sociodemographic characteristics and stress exposure were accounted for, John Henryism was associated with lower odds of fair or poor self-rated health among both groups.

## 1. Introduction

A recent executive summary report revealed that despite being leaders in their communities and making significant contributions to the society and economy of the United States, Black women continue to be underpaid and consistently receive fewer benefits compared to the level of their labor productivity [1], a reality that has persisted since enslavement. These conditions heavily contribute to the elevated health burden observed among this group, and growing evidence demonstrates that Black women face more health challenges than women of other racialized groups. For instance, Black women report disproportionate rates of chronic physical health conditions (e.g., obesity, heart disease, and autoimmune disorders) [2,3,4] and are more than three times as likely to experience maternal mortality compared to White women [5]. Nevertheless, there are additional health challenges that Black women face which may go underexplored. One of those is lower ratings of self-rated health. 

Self-rated health captures perceived health status and general physical functioning [6]. Research has indicated that Black women are more likely to report fair or poor self-rated health [7,8] compared to other populations of women. This is notable for several reasons. Prior studies indicate that self-rated health is a strong predictor of premature mortality, even after accounting for sociodemographic and medical risk factors [6]. Others have shown that self-rated health is a stronger predictor of mortality than physician assessments [9] and current morbidity [10]. Moreover, studies report that self-rated health has predictive validity for numerous physical health indicators and mortality across racial and gender subgroups [6,11]. Thus, given that Black women tend to report lower ratings of their perceived physical health status, there is a need to better understand the factors that shape self-rated health among this group. 

Black women encounter various risk factors that are typically associated with poor self-rated health in the general population; however, Black women also encounter distinct risks across the life course that may contribute to their self-perceptions of physical health. Research suggests that the health risk associated with certain sociodemographic characteristics may be different for Black women. For example, previous scholarship has demonstrated that older Black individuals’ are more likely to report lower ratings of self-rated health compared to their White counterparts, and that their self-rated health tends to decline at a higher rate than younger Black individuals [12,13]. Additionally, Black women report increased exposure to financial strain, racism-related stress, and chronic stress compared to other racial groups [3,4,14], and research shows that these exposures are significantly associated with a range of adverse health outcomes including lower ratings of self-rated health [15,16]. Additionally, stressors such as everyday discrimination and goal-striving stress (i.e., psychological weight of striving for but not achieving upward mobility) have been associated with lower ratings of self-rated health among Black individuals [17,18,19]. While other groups encounter these stressors as well, scholars also note that the stress experiences of Black women are a distinct consequence of a larger system and process grounded in white supremacy, which subjugates and oppresses Black women, particularly within the social context of the United States [20]. Importantly, although stress exposure (i.e., exposure to stressful events or persistent difficulties) has been shown to shape self-rated health among Black women, the consideration of other factors, such as coping styles have been less widely examined [21]. 

Evaluating the role of coping may provide a more comprehensive understanding of how stressful experiences across the life course shape self-rated health among Black women. The Social Stress Paradigm, a prominent theoretical framework in the field of Medical Sociology, indicates that coping is an integral component of the stress process and posits that an individual’s access to a variety of coping resources may influence the ways in which stress ultimately impacts their health [21,22]. Importantly, an individual’s social position determines the types of coping strategies accessible to them and the effectiveness of these coping strategies for health [21,23]. Thus, evaluating the significance of coping may provide a more comprehensive understanding of how stressful experiences across the life course shape self-rated health among Black women. 

While gender-specific forms of coping such as the Superwoman Schema or “Strong Black Women role”, have gained growing attention, far less is known about the impact of other racially relevant forms of coping, including John Henryism [3,4,14,24,25]. One of the few empirically tested constructs that considers the social and cultural experiences of Black Americans [26], John Henryism is defined as persistent, high-effort coping with psychosocial and environmental stressors [24,27]. John Henryism reflects the broader societal, cultural, and historical context that shapes the lived experiences of Black populations navigating racism and capitalism in the U.S. Thus, an individual whose coping style is characterized by “high” John Henryism is more likely to endorse efficient mental and physical stamina, an obligation to work hard, and a focused resolve to achieve [27]. In other words, an individual who engages in high levels of this coping style may be more apt to address stressors and challenges with determination [28]. Conversely, someone whose coping style is characterized by “low” John Henryism is less likely to endorse these characteristics [27]. That is, a person who engages in low levels of John Henryism may be easily overwhelmed by life’s challenges [28]. Thus, while John Henryism is not inherently harmful, research suggests that prolonged engagement in this coping style, particularly within the context of low socioeconomic status (SES) and high stress exposure, may ultimately contribute to heightened health risk [27,29]. 

Although John Henryism is deemed culturally relevant for Black Americans, the original conceptualization of this construct was primarily based on the experiences of Black men. As such, the ways in which this coping style shapes health among Black women has been less clear. For example, only one study to-date has examined the impact of John Henryism on self-reported physical health among Black Americans. Bonham and colleagues [30] found that John Henryism is associated with positive self-rated physical health among Black men, particularly those with high SES levels. However, several gaps limit our understanding of John Henryism’s influence on self-rated health among Black women, which may shed light on the patterns seen among this group.

Firstly, John Henryism has not been extensively examined among women. An individual’s social position shapes the types of coping strategies accessible to them and the effectiveness of these coping strategies for health [21,23]. Although coping resources may be particularly impactful among socially disadvantaged individuals, there has been limited consideration of these processes among Black women. This is especially evident within the literature on John Henryism, as less is known about the potentially divergent mechanisms through which John Henryism may shape the physical health, in particular, self-rated health of Black women. Given this dearth of knowledge, additional research is needed to clarify the health consequences of John Henryism among Black women. Clarifying these processes will help to identify at-risk subgroups, while also distinguishing effective points of intervention in efforts to offset subsequent health risks for Black women. 

Secondly, the role of ethnicity in shaping John Henryism and its impact on health among Black Americans and women remains unclear. Most health research, including work focused on John Henryism, considers Black women as a monolith and does not explicitly evaluate the role of ethnicity. However, there are several reasons why consideration of ethnicity would advance our understanding of how John Henryism shapes Black women’s self-rated health. Ethnicity has a significant influence on health processes that is distinct from racial and cultural influences [31]. Although race and ethnicity are habitually conflated, these constructs are, in fact, theoretically distinct. For instance, race is defined as, “a social construct, a social classification based on phenotype, that governs the distribution of risks and opportunities in our race-conscious society” [31] (p. 300). In contrast, ethnicity refers to “the voluntary grouping of individuals according to shared geographic birth-place and national heritage” [32,33,34] (p. 258). For example, in terms of Black women collectively, this group is considered Black from a racial standpoint in the United States, while individual Black women may vary on ethnic backgrounds (i.e., African American women and Caribbean Black women). Moreover, scholars have also shown that both race and ethnicity predispose individuals to a variety of social stressors, given that these components are indicators of social stratification [31], and that empirically supported predictors, such as gender, age, and SES, vary by race, ethnicity, and cultural influences [31]. 

The social stratification of Black women and how these processes shape health cannot be thoroughly examined without an explicit discussion of slavery. The Trans-Atlantic Slave Trade took place between 1529 and 1850 [35,36,37]. During this time, over 12 million African people, primarily from the coasts of West Africa were kidnapped, ripped away from their loved ones, and forcibly shipped like chattel through the Middle Passage over the Atlantic Ocean [35,37]. These individuals endured hundreds of years of involuntary servitude. Consequently, this process has fostered the intergenerational geographic separation of people of African descent and has produced an unknown degree of trauma, and thus the modern African diaspora [36]. The modern African diaspora is comprised of the millions of individuals of African descent who live in different societies who share a history that is largely but not solely based on racial oppression and the challenges associated with it [36]. Despite these difficulties, people of African descent have been able to recreate their culture(s) in ways that honor their ancestry and lived experiences. Nonetheless, the historical legacies of racial capitalism and exploitation have prompted many people of African descent to relocate to areas that are thought to provide more substantial opportunities for upward mobility [34,38,39]. One place being the United States. 

Collectively, the dearth of research focused on ethnic variations in John Henryism and its health impact among Black women is of particular importance for several reasons. Between the 1960s and 1990s, there was a 25% rise in the Black population within the United States, with Caribbean Black immigrants leading this growth [40]. This population has since grown tremendously, such that today, there are over 4.4 million Caribbean Black immigrants in the United States, and women comprise over 50% of this group [41,42]. Since research shows that immigration processes distinctively shape, and sometimes alter the life chances and health of those who relocate, as well as subsequent generations, it is imperative to investigate the health patterns of Caribbean Black individuals in the United States [21,43,44]. For example, studies have documented disparate physical health trends between African American and Caribbean Black women, such that a higher proportion of African American women report increased odds of obesity, regular alcohol consumption, and lower ratings of self-rated health compared to Caribbean Black women [45,46]. 

On one hand, researchers have noted that factors such as selection processes, resilience, and cultural practices may in fact provide health protection for Caribbean Black individuals from social and environmental stressors [47]. On the other hand, given the existence of the modern African Diaspora, there is a level of shared consciousness and linked fate among people of African descent, which may be of particular relevance in determining the extent to which John Henryism shapes health among Black women and ethnic subgroups of this population. Nevertheless, this area of research has not been heavily explored among African American and Caribbean Black women. Moreover, taking into account the historical, racial, and sociopolitical context of the construct of John Henryism, and the understanding that life experiences and socialization processes may uniquely shape the development of unique coping tools among African American and Caribbean Black women, it is possible that John Henryism’s relationship to self-rated health among Black women is shaped by ethnicity. Furthermore, given that high-effort coping gives individuals a way to mentally persevere through challenges [29], and self-rated health is tantamount to an individual’s mental understanding of their physical health status, it is possible that John Henryism operates in similar ways with respect to how Black women of different ethnicities perceive their health. However, this has not been widely examined.

To address these gaps, the present study (1) assessed the sociodemographic and stress-related correlates of self-rated health; (2) examined the direct association between John Henryism and self-rated health; and (3) evaluated the association between John Henryism and self-rated health after accounting for sociodemographic characteristics and stress exposure. These associations were assessed among African American and Caribbean Black women collectively and explored among Caribbean Black women specifically. 

## 2. Materials and Methods

This study was a secondary analysis of de-identified data from the National Survey of American Life (NSAL 2001–2003), which was collected between 2001 and 2003. The NSAL was a component of the Collaborative Psychiatric Epidemiology Surveys [48,49]. The purpose of the NSAL study was to assess and understand the complexity of mental health disorders among subsamples of Black and non-Hispanic White populations in the United States [48]. The NSAL used multi-stage probability methods to obtain complex samples. The sample for the study included adults in the United States who spoke English and were 18 years of age or older. The study did not include individuals who would be considered institutionalized (i.e., incarcerated individuals, those in the military, people experiencing houselessness) [48]. The study disaggregated individuals according to ethnicity to include African Americans (*n* = 3570), Caribbean Black individuals (*n* = 1621), and non-Hispanic White individuals (*n* = 891), totaling 6082 individuals [48]. African American individuals were those who did not identify as having Caribbean ancestry. Caribbean Black individuals were those who self-identified as Black and had West Indian or Caribbean ancestry or reported that their parents or grandparents were from the Caribbean. Samples for the Black populations were national. The sample of White individuals represented those who resided in census tracts in which the Black population was 10% or more [48]. Nevertheless, all racial and ethnic groups included in the study sample were similar to nationally representative samples in terms of income, gender, marital status, and other factors [48]. This sampling technique was also used to achieve racial and ethnic concordance between interviewers and participants so that that the race and ethnicity of an interviewer would match that of the respondent [48]. Majority of the interviews took place face to face using a computer-assisted device, with approximately 14% taking place either completely or partially via telephone [48]. Participants received an incentive of $50 following the interview. The NSAL included wide-ranging assessments of social and neighborhood conditions, psychosocial risk and protective factors, psychological distress, and stress exposure [48]. NSAL respondents were also asked to complete a mail-in survey, after initial data collection. The mail-in survey is where key study variables (i.e., John Henryism) were assessed. The NSAL was conducted in accordance with the 1964 Declaration of Helsinki and its later amendments. NSAL data collection was approved by the Institutional Review Board at the University of Michigan. Additional information about the NSAL can be found elsewhere [48,49,50]. 

### 2.1. Analytic Sample

While the full NSAL sample was 6082, only individuals who completed the mail-in survey where key study variables can be found (i.e., John Henryism) were included in this study (*n* = 3438). Given that the population of interest for this study was Black women, White individuals and men of all races were excluded (*n* = 1670). This exclusion resulted in a sample of 1768 Black women. Next, individuals missing on key study variables were excluded (*n* = 188) for complete case analysis. Sensitivity analyses were conducted to identify potential distinctions between these excluded individuals (i.e., those missing on key study variables) and the remaining sample; results indicated that individuals who were missing were statistically similar in characteristics to those in the complete sample. The final analytic sample for this study included 1580 Black women, of which 1209 were identified as African American and 371 as Caribbean Black. Approximately 80% of the Caribbean Black women in the analytic sample were born in the United States, which indicates these participants would classify as second-generation immigrants or higher.

### 2.2. Measures

*Outcome Variable.* Self-Rated Health (SRH) was assessed using a single item in the NSAL. Participants were asked how they would rate their health in general. The response options for this item were on a Likert scale ranging from 1 = poor to 5 = excellent. Consistent with previous research [51,52], the self-rated health answer choices were dichotomized: (0) Very Good/Good/Excellent SRH (reference category), and (1) Fair/Poor SRH. 

*Predictor Variables.* Three separate indicators of stress exposure were used. Chronic Stress was assessed in the NSAL using a 10-item checklist with a prompt of, “Over the past month or so, have you…”, with items including ‘had health problems?”, “had family or marriage problems?”, and “had problems with the police?”. Response options were “yes” or “no”. For each respondent, answers endorsing the items were summed, such that higher scores indicated increased exposure to chronic stress. 

Everyday Discrimination was assessed in the NSAL using the Everyday Discrimination Scale developed by Williams and colleagues [53]. This scale includes 10-items (α = 0.88) that assess different experiences of perceived discrimination. The overall prompt was for respondents to identify how frequent each of the ten items had occurred for them in their daily lives over the past year. A sample item is, “People behave as if they think you are not honest” [53]. Respondents were asked to provide answers on a Likert scale of 1 “never” to 6 “almost daily” [53]. Items were summed such that higher scores indicated a higher frequency of discriminatory events. 

Goal-striving stress was assessed in the NSAL using four items that were designed to measure the discrepancy between an individual’s aspirations for a goal, and their achievement, weighted by the chances of achieving that goal, and the level of disappointment that would occur if an individual were to not achieve the goal [18]. The equation for goal-striving stress is the following: (Aspirations − Achievement) * (Chances * Importance). From this equation, a continuous measure was created such that higher scores indicated higher goal-striving stress. Individuals who indicated that they did not have any aspirations were given a goal-striving stress score of zero. To account for the non-normal distribution of this variable, it was categorized based on the 25th and 75th percentiles. The categories were: (1) low goal-striving stress (reference category), (2) moderate goal-striving stress, and (3) high goal-striving stress. Given that the distribution of goal-striving stress was distinct for Caribbean Black women, goal-striving stress remained continuous for this group.

*Coping.* John Henryism was assessed in the NSAL using the validated John Henryism Active Coping Scale (JHAC-12). This 12-item, validated scale (α = 0.82) was developed by Dr. Sherman James [24], and it asks respondents to identify how true each item or statement was for them. A sample item is “I’ve always felt that I could make of my life pretty much what I wanted.” (James et al., 1983). Response options ranged from 1 “completely true” to 5 “completely false”. Items were reverse-coded and summed, such that higher scores indicated increased levels of John Henryism. Given the central focus of the John Henryism construct in this study, individuals who were missing on any of the 12 items were excluded from the analysis. To account for potential threshold effects [28,54], and the non-normal distribution of this variable, John Henryism scores were categorized based on the 25th and 75th percentiles, resulting in the following coding: (1) low John Henryism (reference category), (2) moderate John Henryism, and (3) high John Henryism. A separate analysis indicated that two items of the JHAC-12 scale did not capture the construct of John Henryism among Caribbean Black women. Thus, a John Henryism variable was created for this group that excluded those two items (α = 0.80). The John Henryism variable for Caribbean Black women was also categorized based on the 25th and 75th percentiles. The three categories were: (1) low John Henryism (reference category), (2) moderate John Henryism, and (3) high John Henryism. 

*Sociodemographic Characteristics.* Age was assessed in the NSAL through asking respondents to provide their age in years at the time of the study [48] and was measured continuously in this study.

Socioeconomic Status (SES) was assessed in the NSAL from respondents via multiple indicator variables, including educational attainment and household income. Educational attainment was assessed continuously with respondents indicating the highest number of years of education they had achieved. While most values were numeric, the lowest and highest response options were categorized as “4 or less” and “17 or more”. Household Income was measured continuously in the NSAL, with respondents providing their household income in a dollar amount [48,55]. An SES score was generated for each participant. To create this, values for educational attainment and household income were first standardized and then the scores for these two dimensions (educational attainment and household income) were summed for each participant. This process created a composite or index of SES that represents the number of standard deviations higher or lower each participant’s SES level is relative to the sample’s mean SES [56]. Higher scores indicated higher SES. By weighting educational attainment and household income equally, this approach provided a more complete evaluation of SES [57]. Additionally, this measurement of SES may more effectively depict an individual’s placement in a socially stratified society, which is based on their concurrent positions in various social locations [56]. 

### 2.3. Statistical Analyses

Weighted proportions for all categorical variables were estimated for the full sample. Weighted means, standard deviations, and variable ranges for continuous variables for the full sample are also presented. Logistic regression was used to estimate odds ratios and 95% confidence intervals to (1) assess the sociodemographic and stress-related correlates of self-rated health; (2) examine the direct association between John Henryism and self-rated health; and (3) evaluate the association between John Henryism and self-rated health after accounting for sociodemographic characteristics and stress exposure. Stratified analyses were conducted for each aim. Analyses of “Black women” refer to the collective sample, which included both “African American” and “Caribbean Black”-identified women, whereas analyses for “Caribbean Black women” refers only to women identified as Caribbean Black. The following modeling strategy was used: Model 1 included self-rated health regressed on chronic stress, everyday discrimination, goal-striving stress, age, and SES; Model 2 included self-rated health regressed on John Henryism only; Model 3 included self-rated health regressed on John Henryism, chronic stress, everyday discrimination, goal-striving stress, age, and SES. For analyses conducted with the collective group of Black women (African American and Caribbean Black women jointly), each model also included ethnicity as a variable.

Statistical analyses were conducted using Stata 17.0. To account for the complex sampling design of the NSAL, to ensure that variances were accurate, and estimates can be generalized to the population level, appropriate survey weights were used.

## 3. Results

### 3.1. Sample Characteristics of Black Women

Weighted means and proportions were calculated to better understand the distribution of Self-Rated Health, John Henryism, stress exposure, and sociodemographic characteristics among Black women. Table 1 shows the distribution of these sample characteristics. Approximately 78% percent of the sample reported very good/good/excellent self-rated health, while 22% reported fair/poor self-rated health, which indicates that most Black women rated their physical health as very good/excellent. While not a majority, a large portion of the sample (47.42%) reported moderate John Henryism, while 20.13% reported low John Henryism, and 32.45% reported high John Henryism. While the range for chronic stress was 0 to 8, most women reported about two chronic stressors (M = 1.86; SD = 1.93). The range for everyday discrimination was 0 to 60, most women reported an average score of almost 21 (M = 20.88; SD = 8.55). Most of the sample (36.27%) reported moderate goal-striving stress, while 30.71% reported low goal-striving stress, and 33.01% reported high goal-striving stress. These findings demonstrate that most women reported relatively low stress exposure. Although the range for age was 18 to 93, most women were about 42 years of age (M = 42.29; SD = 20.06), which illustrates that women were about middle-aged. While the range for SES was −4.45 to 6.93, most women reported an SES score of −0.08 (M = −0.08; SD = 1.96), which indicates that most women were of lower SES. Overall, Black women reported very good/good/excellent self-rated health, engaged in moderate levels of high-effort coping, reported fairly low to moderate levels of stress exposure, with the exception of goal-striving stress, were middle-aged, and of lower SES.

### 3.2. Sample Characteristics of Caribbean Black Women

Table 2 shows sample characteristics for Caribbean Black Women. Roughly 86% of Caribbean Black women reported very good/good/excellent self-rated health, while about 14% reported fair/poor self-rated health. Overall, a large proportion of Caribbean Black women (52.40%) reported moderate John Henryism, while 18.21% reported low John Henryism, and 29.39% reported high John Henryism, which indicates that most Caribbean Black women engaged in moderate levels of high-effort coping. Most Caribbean Black women reported relatively low stress exposure. While the range for chronic stress was 0 to 8, most women reported about two (M = 1.87; SD = 3.85), which indicates that most Caribbean Black women reported low exposure to chronic stress. While the range for everyday discrimination was 0 to 60, most Caribbean Black women reported an average score of 22 on the everyday discrimination scale (M = 22.02; SD = 20.03), which indicates that most Caribbean Black women reported somewhat moderate frequency of exposure to everyday discrimination. While the range for goal-striving stress was 0 to 84, most Caribbean Black women reported about 7 (M = 6.84; SD = 17.04), which indicates that most Caribbean Black women reported low exposure to goal-striving stress. While the range for age was 18 to 92, most Caribbean Black women were about 43 years of age (M = 42.65; SD = 33.08), which indicates that most Caribbean Black women were about middle-aged. While the range for SES was −4.45 to 6.93, most Caribbean Black women reported an SES score of 0.29 (standard deviation, SD = 3.79), which indicates that most Caribbean Black women were of higher SES. Collectively, most Caribbean Black women reported very good/good/excellent self-rated health, engaged in moderate levels of high-effort coping, reported fairly low to moderate levels of stress exposure, were middle-aged, and of higher SES.

### 3.3. Regression Analyses of Black Women

Table 3 shows logistic regression results for aims 1–3 among Black women. Stress exposure and sociodemographic characteristics were associated with self-rated health among Black women (Model 1). More specifically, chronic stress and goal-striving stress were significantly associated with self-rated health among this group. For every one-unit increase in exposure to chronic stress, Black women reported a 43% increase in their odds of reporting fair or poor self-rated health, all else equal (AOR = 1.43; 95% CI = 1.30–1.59; *p* < 0.001). Additionally, compared to Black women who reported low GSS, those with high GSS reported a little over twice the odds of fair or poor self-rated health, all else equal (AOR = 2.06; 95% CI = 1.29–3.28; *p* < 0.01). Both age and SES were significantly associated with self-rated health. For every one-unit increase in age, Black women reported a 3% increase in odds of fair or poor self-rated health, all else equal (AOR = 1.03; 95% CI = 1.02–1.04; *p* < 0.001). Conversely, for every one unit increase in SES, Black women reported a 33% decrease in odds of fair or poor self-rated health, all else equal (AOR = 0.67; 95% CI = 0.58–0.78; *p* < 0.001). John Henryism was not directly associated with self-rated health (Model 2). Nevertheless, after sociodemographic chracteristics and stress exposure were accounted for (Model 3), high John Henryism was found to be associated with lower odds of fair/poor self-rated health. Compared to Black women engaged in low John Henryism, those engaged in high John Henryism reported a 47% decrease in their odds of fair/poor self-rated health, all else equal (AOR = 0.53; 95% CI = 0.31–0.91; *p* < 0.05). In other words, differences in sociodemographic characteristics and stress exposure might explain or account for the association between John Henryism and self-rated health among Black women. 

### 3.4. Regression Analyses of Caribbean Black Women

Table 4 illustrates logistic regression results for aims 1–3 among Caribbean Black women. Stress exposure and sociodemographic characteristics were significantly associated with self-rated health among Caribbean Black women (Model 1). Chronic stress was the only stressor significantly associated with self-rated health among this group. For every one-unit increase in exposure chronic stress, Caribbean Black women reported a 52% increase in their odds of fair or poor self-rated health, all else equal (AOR = 1.52; 95% CI = 1.14–2.02; *p* < 0.01). In terms sociodemographic characteristics both age and SES were significantly associated with self-rated health. For every one-unit increase in age, Caribbean Black women reported a 4% increase in their odds of fair or poor self-rated health, all else equal (AOR = 1.04; 95% CI = 1.01–1.07; *p* < 0.01). In terms of SES, for every one-unit increase in SES, Caribbean Black women reported a 33% decrease in their odds of fair or poor self-rated health, all else equal (AOR = 0.67; 95% CI = 0.47–0.95; *p* < 0.05). John Henryism was not directly associated with self-rated health among Caribbean Black women (Model 2). However, after accounting for sociodemographic characteristics and stress exposure, high John Henryism was significantly associated with self-rated health among this group (Model 3). Compared to Caribbean Black women engaged in low John Henryism, those engaged in high John Henryism reported a 65% decrease in their odds of fair or poor self-rated health, all else equal (AOR = 0.35; 95% CI = 0.13–0.91; *p* < 0.01). This result suggests that if all Caribbean Black women shared the same age, SES, and exposure to chronic stress, everyday discrimination, and goal-striving stress, we would see this association between high John Henryism and lower odds of fair/poor self-rated health among this population.

## 4. Discussion

Despite tremendous contributions to the workforce and overall economy within the United States, Black women continue to disproportionately experience poor physical health outcomes (e.g., obesity, heart disease, and autoimmune disorders). An additional challenge is poor self-rated health. Prior research has shown that Black women are more likely to report fair or poor self-rated health than other racialized groups of women [7,8]. This is worthy of concern given that self-rated health is a predictor of premature mortality, and a stronger indicator of current morbidity and subsequent mortality than physician assessments [9,10]. Moreover, Black women experience distinct risk factors across the life course that shape their likelihood of reporting fair or poor self-rated health. For example, sociodemographic factors and increased exposure to social stress such as financial strain, racism-related stress, and chronic stress significantly contribute to adverse health for Black women, including fair/poor ratings of self-rated health [15,16]. Nevertheless, there has been limited consideration in terms of examining the role of other factors, such as coping, in shaping self-rated health among this group. While there is a growing body of literature examining gender-specific forms of coping such as Superwoman Schema, much less has been evaluated about the impact of other culturally relevant forms of coping, such as John Henryism [3,4,14,24,25]. Moreover, although the role of ethnicity in shaping health processes has received growing attention in the literature [42,47,58], less is known about how this factor shapes John Henryism and health processes among Black women specifically. Thus, the purpose of this study was to examine the impact of sociodemographic characteristics, stressors, and John Henryism on self-rated health among African American and Caribbean Black women. There were several findings that assisted with clarifying the links between stress, coping, and self-rated health among ethnic subgroups of Black women.

First, chronic stress, age, and SES were significantly associated with self-rated health among African American and Caribbean Black women both collectively and among Caribbean Black women specifically. While increases in chronic stress and age were associated with lower ratings of self-rated health, increases in SES were associated with higher ratings of self-rated health. Notably, neither everyday discrimination nor goal-striving stress were associated with self-rated health among African American or Caribbean Black women. These findings align with recent work from Erving [46], that examined the role of stress exposure in shaping physical health among older African American and Caribbean Black women. This study found that chronic stress was a significant predictor of self-rated health among older African American and Caribbean Black women, while everyday discrimination was not [46]. A key difference is that this study’s sample of Caribbean Black women were aged 50 and older, primarily foreign-born, and accounted for SES, while the present study’s population of Caribbean Black women were primarily US-born, aged 18–92 and assessed the predictive capacity of SES. Despite these differences, findings from both studies suggest it is possible that similar stressors may shape self-rated health across the life course among African American and Caribbean Black women, irrespective of nativity status. Nevertheless, additional research on this topic is needed to examine this further.

Second, while John Henryism was not directly associated with self-rated health among either group, after accounting for sociodemographic characteristics and stress exposure, John Henryism was associated with lower odds of fair or poor self-rated health among African American and Caribbean Black women both collectively and among Caribbean Black women specifically. This result suggests that if all African American and/or Caribbean Black women shared the same age, SES, and exposure to chronic stress, everyday discrimination, and goal-striving stress, we would see this association between high John Henryism and lower odds of fair or poor self-rated health among these populations. In other words, differences in these factors might explain or account for this relationship among both groups. This finding aligns with previous work that examined the role of John Henryism in shaping self-rated health among African American men. Bonham and colleagues [30] found that men who were older reported lower ratings of self-rated health, while those who reported high John Henryism reported higher ratings of self-rated health. However, the study by Bonham and colleagues accounted for sociodemographic characteristics and not stress exposure. 

Whereas Social Stress Theory emphasizes the role of coping in shaping health among populations, this theory also underscores the necessity of accounting for sociodemographic characteristics and stress exposure to gain a better picture of how coping shapes health [21,59], which is what was found for self-rated health among the sample. After accounting for sociodemographic factors and stress exposure, high John Henryism was, in fact, associated with lower odds of fair or poor self-rated health among African American and Caribbean Black women. In other words, once forms of social stratification and stress exposure were taken into consideration, we see that high John Henryism is protective or served as a resource for African American and Caribbean Black women’s’ self-perceptions of their physical health status. This may be the case because sociodemographic characteristics and stress exposure shape coping resources [21,59], which ultimately influence physical health. Considering that high-effort coping provides individuals with the mental fortitude to push through challenges [29], and self-rated health is essentially someone’s mental perception of their physical health status, it is possible that John Henryism also taps into similar processes in terms of how African American and Caribbean Black women perceive their health. For example, if someone endorses high levels of high-effort coping, they are mentally pushing themselves to persevere through challenges, which likely provides them with endurance to navigate these circumstances. In turn, these individuals are probably less likely to perceive their physical health status as fair or poor because they feel good due to exerting a sense of control over difficulties. Collectively, these findings suggest that to clarify the physical significance of John Henryism among Black women, it is crucial to account for forms of social stratification and stress exposure. Failure to do so obscures the true influence of John Henryism on self-rated health among African American and Caribbean Black women and stifles possibilities for effective interventions to promote population health among these groups.

These findings also beg the question, “why might African American and Caribbean Black women share experiences in terms of John Henryism, self-rated health, and the factors that shape this association”?. Research has shown that to a certain extent, by the second-generation, health patterns of Caribbean Black immigrants who migrate start to mirror that of the destination area [60]. Given that most Caribbean Black women in the sample were second-generation or higher, this is particularly relevant. Prior work conducted in this area has demonstrated that experiences of racism, and pressure to assimilate by white individuals and other racial and ethnic immigrant groups is extremely impactful for (Caribbean) Black immigrants [40]. More specifically, researchers acknowledge that these stark contradictions are most felt by Caribbean Black immigrants and their children, given that the process for assimilation in the U.S. for them connotes being categorized with African Americans, who have been historically marginalized [34,61,62,63]. Given these experiences, second-generation Caribbean Black immigrants often report that they occupy a unique space of “in-betweenness” because they do not always ascribe to their first-generation parents’ worldview on a variety of topics [40]. Due to a lack of blatant signifiers of their immigrant status, this group is also often assumed to be African American, which significantly impacts how they navigate American society (Lorick-Wilmot 2014). To this end, it is very much possible that due to these complex factors and feelings of “in-betweenness”, overtime in so many ways, Caribbean Black women learn to navigate the United States context as African American women do. 

Although this study provides significant contributions to the literature, there are several limitations to consider. First, although the dataset used to conduct analyses is the most comprehensive assessment of mental health and psychiatric disorder among people of African descent, the dataset is 20 years old (NSAL 2001–2003). Relatedly, the social and political landscape has shifted since this data was first collected, which means that the context in which the original study was conducted may not completely align with what is happening today. Forthcoming work assessing the links between John Henryism and physical health among Black women may benefit from more recent data that adequately captures the current sociopolitical context. Additionally, the data used was cross-sectional, which means that findings from this study cannot be used to establish causality. Therefore, future work on this topic would be enhanced with the collection of longitudinal data. The key dependent variable in the present study was a self-report of physical health status, which is subjective, as opposed to a physician-diagnosed physical health outcome. Future work on this topic would benefit from the use of more objective physical health measures. The next set of limitations for the present study primarily focus on participant characteristics. The present study only included individuals who completed the re-interview mail to home survey because this is where key study variables were assessed (i.e., John Henryism and health measures). The implication for this limitation is that the number of participants available for analyses decreased, which may shape the interpretation of findings from this study to the population-level. Prospective research on these topics may be advanced by implementing data collection methods that allot for adjusting the order of survey items and/or inquiries to lower the likelihood of attrition and non-responsiveness from participants, without compromising confidentiality and privacy of participants.

## 5. Conclusions

Notwithstanding limitations, the present study provides significant contributions to the literature. This is one of the first studies to examine the role of John Henryism in shaping self-rated health among Black women. Moreover, this is one of the first studies to evaluate the association between John Henryism and self-rated health among ethnic subgroups of Black women (i.e., African American and Caribbean Black). Findings show that while chronic stress, age, and SES directly shaped self-rated health among both groups, John Henryism alone did not. It was not until stress exposure and sociodemographic characteristics were accounted for that we saw an association between high John Henryism and higher ratings of self-rated health. In other words, this study clarified that to more fully ascertain the association between John Henryism and self-rated health among ethnic subgroups of Black women, it is important to account for differences in sociodemographic characteristics and stress exposure. Future work in this area would be enhanced by examining interactive associations between John Henryism and various stressors to further understand specific mechanisms shaping these associations.

## Figures and Tables

**Table 1:** Sample Characteristics of Black Women, National Survey of American Life (2001–2003), N = 1580.

Characteristic	Mean or %	SD
Self-Rated Health		
Very Good/Good/ Excellent SRH (Ref.)	77.57	
Fair/Poor SRH	22.43	
John Henryism (JH)		
Low JH	20.13	
Moderate JH	47.42	
High JH	32.45	
Stress Exposure		
Chronic Stress [0–8] ^b^	1.86	1.93
Everyday Discrimination [0–60] ^b^	20.88	8.55
Goal-Striving Stress (GSS)		
Low GSS (Ref.)	30.71	
Moderate GSS	36.27	
High GSS	33.01	
Sociodemographic Characteristics		
Age [18–93] ^b^	42.29	20.06
Socioeconomic Status ^a^ [−4.45–6.93] ^b^	−0.08	1.96

Note: Ref. = Reference Category; weighted means and percentages reported; ^a^ = index; ^b^ = range; SD = standard deviation.

**Table 2:** Sample Characteristics of Caribbean Black Women, National Survey of American Life (2001–2003), N = 371.

Characteristic	Mean or %	SD
Self-Rated Health (SRH)		
Very Good/Good/ Excellent SRH (Ref.)	85.84	
Fair/Poor SRH	14.16	
John Henryism (JH)		
Low JH	18.21	
Moderate JH	52.40	
High JH	29.39	
Stress Exposure		
Chronic Stress [0–8] ^b^	1.87	3.85
Everyday Discrimination [0–60] ^b^	22.02	20.03
Goal-Striving Stress [0–84] ^b^	6.84	17.40
Sociodemographic Characteristics		
Age [18–92] ^b^	42.65	33.08
Socioeconomic Status^a^ [−4.45–6.93] ^b^	0.29	3.79

Note: Ref. = Reference Category; weighted means and percentages reported; ^a^ = index; ^b^ = range; SD = standard deviation.

**Table 3:** Logistic Regression Examining the Association Between John Henryism and Self-Rated Health among Black Women, National Survey of American Life (2001–2003), N = 1580.

Characteristic	Model 1	Model 2	Model 3
John Henryism			
Low JH (Ref.)			
Moderate JH		0.86 [0.60–1.23]	0.71 [0.43–1.18]
High JH		0.66 [0.42–1.05]	0.53 * [0.31–0.91]
Stress Exposure			
Chronic Stress	1.43 *** [1.30–1.59]		1.44 *** [1.31–1.60]
Everyday Discrimination	1.01 [0.99–1.03]		1.01 [0.99–1.03]
Goal-Striving Stress (GSS)			
Low GSS (Ref.)			
Moderate GSS	1.50 [1.00–2.27]		1.50 [1.00–2.26]
High GSS	2.06 ** [1.29–3.28]		2.11 ** [1.33–3.33]
Sociodemographic Characteristics			
Age	1.03 *** [1.02–1.04]		1.03 *** [1.02–1.04]
SES	0.67 *** [0.58–0.78]		0.67 *** [0.58–0.77]
Ethnicity			
African American (Ref.)			
Caribbean Black	0.59 [0.34–1.00]		0.60 [0.36–1.01]
Intercept	0.02 *** [0.01–0.05]	0.35 *** [0.25–0.50]	0.03 *** [0.01–0.07]
F-Statistic	-	1.64	2.86
df	-	(2, 56)	(2, 56)

Note: Adjusted Odds Ratios (AOR) Reported. * *p* < 0.05; ** *p* < 0.01; *** *p* < 0.001 (two-tailed tests; F-Statistic = Adjusted Wald Test (joint-test); df = degrees of freedom.

**Table 4:** Logistic Regression Examining the Association Between John Henryism and Self-Rated Health among Caribbean Black Women, National Survey of American Life (2001–2003), N = 371.

Characteristic	Model 1	Model 2	Model 3
John Henryism			
Low JH (Ref.)			
Moderate JH		0.51 [0.14–1.89]	0.53 [0.18–1.54]
High JH		0.50 [0.17–1.48]	0.35 ** [0.13–0.91]
Stress Exposure			
Chronic Stress	1.52 ** [1.14–2.02]		1.57 ** [1.14–2.18]
Everyday Discrimination	0.96 [0.91–1.01]		0.96 [0.91–1.01]
Goal-Striving Stress (GSS)	1.04 [1.00–1.07]		1.04 * [1.01–1.07]
Sociodemographic Characteristics			
Age	1.04 ** [1.01–1.07]		1.04 ** [1.01–1.07]
SES	0.67 * [0.47–0.95]		0.64 * [0.45–0.91]
Intercept	0.01 *** [0.00–0.08]	0.28 ** [0.11–0.70]	0.02 ** [0.00–0.14]
F-Statistic	-	0.87	2.47
df	-	(2, 25)	(2, 25)

Note: Adjusted Odds Ratios (AOR) Reported. * *p* < 0.05; ** *p* < 0.01; *** *p* < 0.001 (two-tailed tests; F-Statistic = Adjusted Wald Test (joint-test); df = degrees of freedom.

## Data Availability

Restrictions apply to the availability of these data. Data was obtained from the Inter-university Consortium for Political and Social Research (ICPSR) at the University of Michigan.
